# Effect of Chemical Structure and Degree of Branching on the Stability of Proton Exchange Membranes Based on Sulfonated Polynaphthylimides

**DOI:** 10.3390/polym12030652

**Published:** 2020-03-12

**Authors:** Chunmei Gao, Jiale Chen, Boping Zhang, Lei Wang

**Affiliations:** 1 College of Chemistry and Environmental Engineering, Shenzhen University, Shenzhen 518060, China; 2 Shenzhen Key Laboratory of Polymer Science and Technology, College of Materials Science and Engineering, Shenzhen University, Shenzhen 518060, China; 3 Guangdong Research Center for Interfacial Engineering of Functional Materials, Shenzhen University, Shenzhen 518060, China

**Keywords:** proton exchange membrane (PEM), sulfonated polynaphthylimides (SPI), chemical stability, branch structure

## Abstract

Hydrolytic stability and oxidative stability are the core properties of sulfonated polynaphthylimides (SPIs) as proton exchange membranes. The chemical structure of SPIs directly influences the performance. Herein, three different series of branched SPIs were designed and prepared using 1,3,5-tris (2-trifluoromethyl-4-aminophenoxy) benzene as a trifunctional monomer and three non-sulfonated diamine monomers, such as 4,4′-oxydianiline (ODA), 2,2-bis[4-(4-aminophenoxy)phenyl]hexafluoropropane (6FODA), and 4,4′-(9-fluorenylidene)dianiline (BFDA). The effect of the chemical structure and degree of branching on SPIs properties is discussed. The results showed that by controlling the chemical structure and degree of branching, the chemical stability of SPIs changed significantly. SPI-6FODA with two ether linkages and a hydrophobic CF_3_ group has higher hydrolytic stability than SPI-ODA with only one ether linkage. In addition, with the increase of the introduced B_3_ monomer, the oxidation stability of SPI-6FODA has been greatly improved. We successfully synthesized SPIs with a high hydrolytic stability and oxidative stability.

## 1. Introduction

Facing the depletion of oil resources and the increasing environmental pollution caused by fossil fuels, proton exchange membrane fuel cells (PEMFC) have been recognized as one of the clean energy devices in the future [1,2,3,4,5,6,7]. Proton exchange membrane (PEM) is the key part of a PEMFC system, which has been one of the research hotspots in this field in the past few decades [6,8]. Perfluorosulfonic acid polymer membrane, such as Nafion [9,10,11,12], is the universal material for commercial PEM application because of its excellent stability and high ionic conductivity at a fully hydrated condition. However, perfluorosulfonic acid membranes have some drawbacks, such as high cost, high fuel crossover, low operation temperature, and big deformation under fuel cell operation [13,14]. Over the past few decades, hundreds of low-cost sulfonated polymers, such as sulfonated polyether-ether-ketone [15,16,17,18,19,20], sulfonated polyether sulfone [21,22,23,24,25,26,27,28,29,30,31], and sulfonated polynaphthylimides with different chemical structures, have been synthesized for PEMFCs applications [28,29,32]. Among them, sulfonated polynaphthylimides (SPIs) have attracted considerable attention because of their low cost, excellent physical properties, high methanol gas barrier ability, high thermal stability, and easy-to-adjust chemical structure [33,34,35,36]. However, the unsatisfied stability of SPI membrane in a fuel cell under high temperature and high humidity conditions cannot be ignored [37,38,39]. This is because the amide bonds with low electron cloud density on the imide ring are easily attacked by water molecules causing hydrolysis [40]. Studies have shown that the hydrolytic stability of sulfonated polyimide PEM can be improved by changing the monomer structure to increase the electron cloud density of the nitrogen atom and the carbonyl carbon atom. For an aromatic amino monomer, the introduction of an electron donating group, such as an ether bond, can increase the electron cloud density of the nitrogen and carbonyl carbon atoms to achieve higher hydrolytic stability [41]. The SPI synthesized from 4,4′-bis(4-aminophenoxy)biphenyl as a non-sulfonated diamine monomer remains intact after being placed in water at 130 °C for 500 h [42,43]. Nevertheless, the introduced electron donating groups, such as aliphatic and ether linkages, were easily attacked by hydroxyl radicals resulting in poor oxidative stability during the operation of the fuel cell [44,45]. Therefore, it is still a tricky endeavor to put a tradeoff between hydrolytic stability and oxidative stability of polyimide.

In recent years, branching has been widely used in proton exchange membrane modification. Recently, our group found that branching is one of the effective approaches to improve oxidative stability of sulfonated poly(fluorenyl ether ketone sulfone)s, sulfonated block poly(arylene ether)s, etc. [46,47,48,49,50,51,52]. At the same time, the introduction of branched structures also significantly increased the proton conductivity of the membranes. 

Based on the above statement, in order to obtain SPI based membranes with a high performance, the effect of the polymer’s scaffold and the degree of branching on the properties was investigated in this study. Firstly, three different non-sulfonated diamine monomers, such as 4,4′-oxydianiline (ODA) with one ether bond, 2,2- bis[4-(4-aminophenoxy)phenyl]hexafluoropropane (6FODA) with two ether bonds and hydrophobic -CF_3_, and 4,4′-(9-fluorenylidene)dianiline (BFDA) large steric hindrance and 1,3,5-tris (2-trifluoromethyl-4-aminophenoxy) benzene with ether bond and -CF_3_ group as a trifunctional monomer were selected to synthesize three series of branched SPIs with different degrees of branching. Then, these three series of branched SPIs with different branching degree were fabricated as proton exchange membranes, and their corresponding properties were characterized. The effects of different non-sulfonated diamine monomers and introduced branched structures on the performance of SPIs as a proton exchange membrane were studied.

## 2. Materials and Methods 

### 2.1. Materials

1,4,5,8-naphthalenetetra-carboxylic dianhydride (NTDA), phloroglucinol, 2-Fluoro-5-Nitrobenzotrifluoride, hydrazine hydrate, 10% palladium on charcoal (Pd/C), m-cresol, triethylamine (TEA), dimethyl sulfoxide (DMSO), dimethylacetamide (DMAC), 4,4′-oxydianiline (ODA), 2,2-bis[4-(4-aminophenoxy)phenyl]hexafluoropropane (6FODA), and 4,4′-(9-fluorenylidene)dianiline (BFDA) were purchased from Energy Chemical Co., Shanghai, China. 4,4′-Diamino-2,2′-biphenyldisulfonic acid (DAPS, 75%) was purchased from Dibo Chemical Reagent Co., Shanghai, China. Unless otherwise noted, all other reagents were used directly. DMSO, DMAC, m-cresol, toluene, and TEA were dried using 4-Å molecular sieves prior to use. DAPS was recrystallized using triethylamine and hydrochloric acid. ODA, 6FODA, and BFDA were dried in a vacuum at 80 °C for 6 h before use, and NTDA was dried in a vacuum at 120 °C for 24 h before use. 

### 2.2. Synthesis of B_3_ Monomer (TFAPOB)

**Scheme 1:**
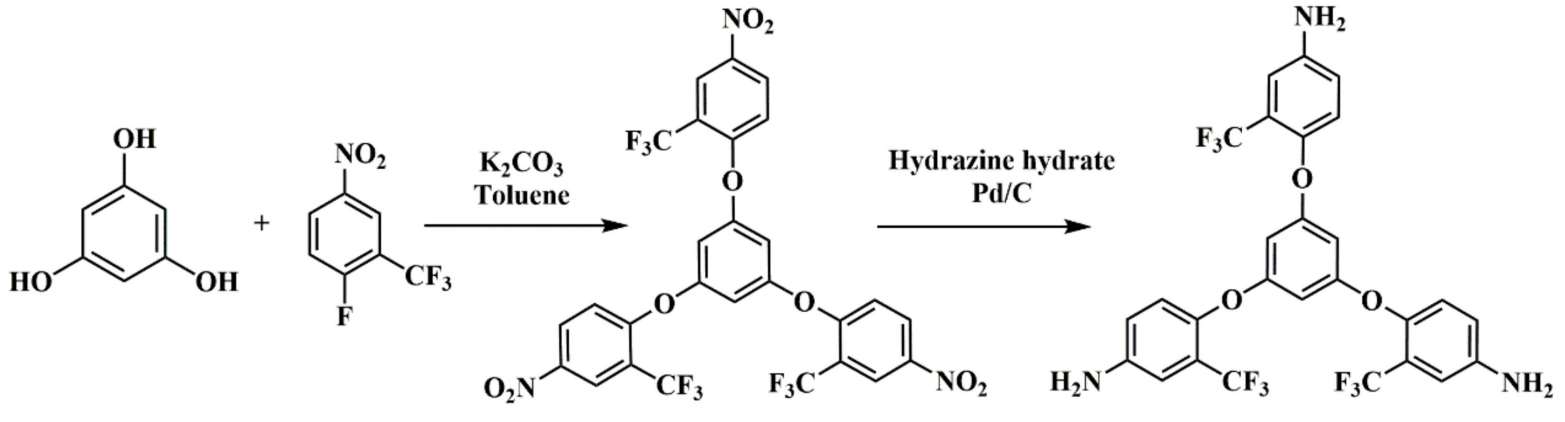
Synthesis route of B_3_ monomer.

1,3,5-Tris(2-trifluoromethyl-4-aminophenoxy) benzene (TFAPOB) was synthesized by using the two-step approach. The synthetic route is shown in Scheme 1. The intermediate 1,3,5-tris(2-trifluoromethyl-4-nitrophenoxy) benzene was synthesized in the first step. Phloroglucinol (0.63 g, 5 mmol), 2-fluoro-5-nitrobenzotrifluoride (5.225 g, 25 mmol), and K_2_CO_3_ (2.07 g, 15 mmol) were added into a 50 mL three-necked flask equipped with nitrogen inlet, magnetic stirrer, Dean–Stark trap and condenser, and DMAC (25 mL) and toluene (5 mL) were the solvents. The reaction mixture was heated at 140 °C to remove the water. After complete elimination of water, the temperature of the reaction increased to 175 °C and refluxed for 10 h. After cooling to room temperature, the mixture was slowly poured into 500 mL of methanol to obtain the intermediate and was dried in a vacuum. 

In the second step, the synthesized 1,3,5-tris(2-trifluoromethyl-4-nitrophenoxy)benzene, ethanol, and palladium carbon were added to a three-necked flask equipped with a constant pressure funnel and condenser. The mixture was stirred and fully dispersed under the protection of nitrogen. After heating to 60 °C, hydrazine monohydrate was slowly added with a constant pressure funnel, refluxed at 60 °C for 8 h, and then filtered. Finally, the filtrate was concentrated to 1/3 and then poured into a 30% NaCl solution and stirred vigorously to obtain a flocculated yellow precipitate. After filtration, the precipitates were vacuum dried at 60 °C for 24 h, and the B_3_ monomer was obtained with a yield of 64%.

### 2.3. Synthesis of Sulfonated Polyimide (SPI)

**Scheme 2:**
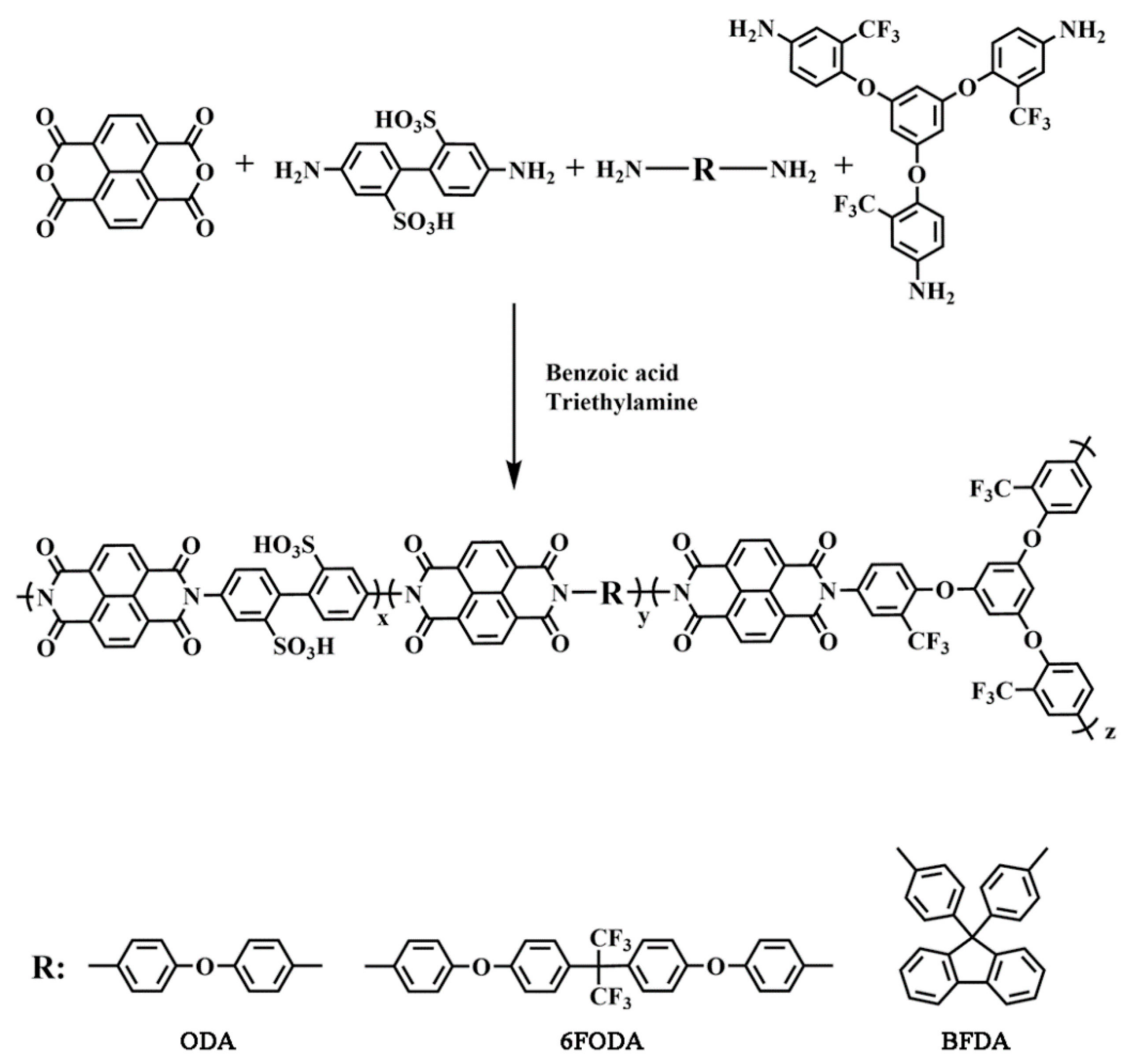
Synthesis route of sulfonated polynaphthylimides.

SPI was synthesized by using dianhydride monomer (NTDA), sulfonated monomer (DAPS), B_3_ monomer (TFAPOB), and three different non-sulfonated diamine monomers (ODA, 6FODA, BFDA). The synthetic route is shown in Scheme 2.

#### 2.3.1. Preparation of Linear SPI-ODA 

A total of 0.344 g (1 mmol) of DAPS, 1 mL of triethylamine, and 5 mL of m-cresol were added into a 50 mL three-necked flask. In N_2_ protection, the reaction mixture was stirred constantly at 80 °C until the DAPS dissolved completely. Next, 0.5 mmol of non-sulfonated diamine monomer (ODA, 0.1 g) was added and stirred continuously until complete dissolution of non-sulfonated diamine monomers. Then, 0.402 g (1.5 mmol) of NTDA and 0.26 g of benzoic acid were added to the above solution. After complete dissolution of NTDA and benzoic acid, the reaction mixture was heated to 80 °C for 4 h and 180 °C for 20 h. After cooling to 80 °C, the mixture was slowly poured into 500 mL methanol to precipitate the polymer. The polymer was washed twice with large amounts of acetone and methanol. The resulting product was dissolved in DMSO and then precipitated in methanol twice and vacuum dried at 120 °C for 24 h. The other liner SPI polymers (SPI-6FODA, SPI-BFDA) were synthesized in a similar manner.

#### 2.3.2. Preparation of Branched SPI

Based on the degree of branching, the required amount of B_3_ monomer was dissolved in m-cresol. Next, the synthesis process of the linear SPI was repeated. When the viscosity of the solution suddenly increased at 180 °C, the mixture was poured into methanol, and the subsequent processing of the linear SPI was repeated.

### 2.4. Preparation of Membranes

A total of 1 g of polymer was stirred in 20 g of DMSO containing 0.5 mL of TEA at 80 °C. After obtaining a clear solution, the solution was filtered using absorbent cotton and cast onto a preheated flat glass plate at 80 °C. After drying for 4 h under normal pressure at 80 °C and heated to 120 °C for 12 h, the resulting membrane was immersed in methanol at 40 °C to completely remove the solvent and then soaked in a 1 mol L^−1^ hydrochloric acid solution for 12 h. Finally, the membranes were washed thoroughly with deionized water. The acidified membrane was fully imidized by immersing it in acetic anhydride:pyridine mixed solution (1:1, v) at 80 °C for 12 h. Finally, the film was immersed in methanol at 50 °C for 12 h to remove the impurities and dried.

### 2.5. Measurements

The thermal gravimetric analysis (TGA) of SPIs were measured with Q50 (TA Instruments, NewCastle, DE, USA) at a heating rate of 10 °C min^−1^ under the protection of N_2_. The Fourier-transform infrared spectra (FTIR) of the SPIs were recorded using a Nicolet 6700 spectrometer (Thermo Scientific, Nicolet, Waltham, MA, USA). ^1^H NMR of the B_3_ and SPIs were recorded using a Bruker AVANCE III 500 MHz NMR instrument. The microstructure of the membranes of SPI-BFDA was tested by Field emission scanning electron microscopy (FESEM, S-3400N). The mechanical property of SPIs was measured with an electromechanical universal testing machine (CMT 4204, MTS systems, Jinan, China) at a stretch rate of 5 mm min^−1^ (the relative humidity was 85%). The SPIs test samples were cut into (15 mm × 4 mm) dumbbell samples.

### 2.6. Water Uptake and Ion Exchange Capacity

Water uptake of the membranes was measured according to the following procedures. The sample was dried in a vacuum oven at 80 °C for 24 h. The dry membranes were weighed and then soaked in deionized water at different temperatures (20 °C or 25 °C, 40 °C, 50 °C, 60 °C, 70 °C, 80 °C) for 2 h. After that, the wet membranes were taken out, and the excess moisture at their surfaces was absorbed by filter paper. Then, their weights were rapidly measured. Water uptake (WU) of the membranes was measured by

(1) $$\mathrm{WU}(\mathrm{wt}.\%)=\frac{W_{\mathrm{wet}}{-W}_{\mathrm{dry}}}{W_{\mathrm{dry}}}\times 100,$$

where W_wet_ and W_dry_ are mass (g) of moist and dry membranes, respectively.

Ion-Exchange Capacity (IEC, mmol g^–1^) was measured by titration. The sample was dried in a vacuum oven at 80 °C for 24 h and weighed. Then, the membrane was immersed in a saturated NaCl solution for 48 h to exchange H^+^ ions with Na^+^ ions. The exchanged H^+^ ions within the solution were titrated with a 0.01 mol L^–1^ NaOH solution using phenolphthalein as an indicator. We calculated the IEC with Equation (2):

(2) $$\mathrm{IEC}(\mathrm{mmol}\;g^{-1})\;=\frac{M_{\mathrm{NaOH}}{\times V}_{\mathrm{NaOH}}}{W_{\mathrm{dry}}},$$

where M_NaOH_ and V_NaOH_ are the molar concentration (mol L^−1^) and volume (mL) of the consumed NaOH, and W_dry_ is the weight of the dried membrane.

The theoretical IEC (IEC_th_) was calculated according to the Equation (3):

(3) $${\mathrm{IEC}}_{\mathrm{th}}(\mathrm{mmol}\;g^{-1})\;=\frac{{n(-\mathrm{SO}}_{3\;}H)}{W_{\mathrm{dry}}},$$

where n(-SO_3_H) represents the mol number of sulfonic groups in reactants, W_dry_ is the theoretical weight of as-prepared membrane, which is equal to total mass (g) of reactants minus the weight of produced water.

### 2.7. Proton Conductivity

The proton conductivity (σ, S cm^–1^) of the membrane was determined by AC impendence spectroscopy (Solartron 1260A) with an oscillating voltage of 10 mV over a frequency range from 100 KHz to 1 Hz. The strip-like film sample (1 cm × 4 cm) was placed in a constant temperature and humidity chamber (98%), and the test temperature ranged from 25 to 80 °C. Proton conductivity of the membrane was calculated by Equation (4)

(4) $$\sigma (S{\mathrm{cm}}^{-1})=\frac{L}{\mathrm{RA}},$$

where σ (S cm^–1^) is the proton conductivity, L is the distance (cm) between two electrodes, R is the measured membrane resistance, and A is the cross-sectional area of the membrane.

### 2.8. Chemical Stability

The oxidation stability of the membrane sample (2 cm × 1 cm) was determined by Fenton’s reagent (30% H_2_O_2_ + 20 ppm FeSO_4_), and the test temperature was 30 °C. The oxidation stability is expressed in terms of its rupture time and complete dissolution time.

The hydrolysis stability of the membrane sample (2 cm × 1 cm) was determined by immersing the deionized water at 80 °C. The hydrolytic stability was expressed as the mass loss of the membrane sample every 24 h.

## 3. Results and Discussion

### 3.1. SPIs Synthesis And Characterization

Both the linear and branched SPIs were synthesized using one-step synthesis. Three SPI series with a 66.7% degree of sulfonation and different branching degrees were synthesized by changing the ratio of different monomer additions. The monomer feed ratio, degree of branching, and sulfonated level of the various SPIs are listed in Table 1.

**Figure 1:**
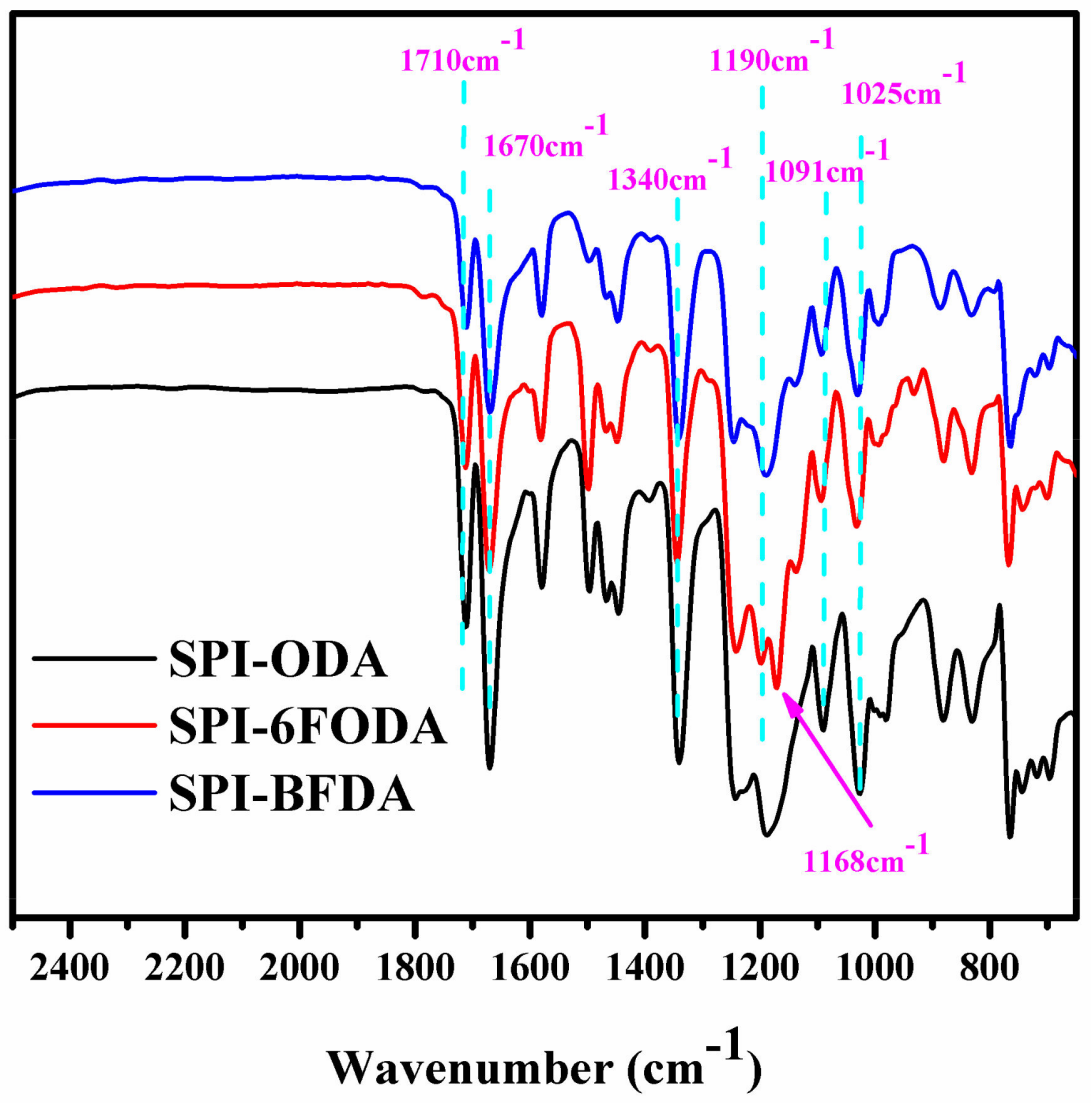
FT-IR spectra of three series of sulfonated polynaphthylimides (SPIs).

Since the highly branched structure is easily cross-linked during the synthesis, the reaction time of the branched polymer is shortened, and the film is finally soaked in a mixed solution of acetic anhydride and pyridine to completely imidize the film. Figure 1 shows the FT-IR spectra of three series of SPIs. The typical absorption bands of the imido ring are found at 1710, 1670, and 1340 cm^−1^. The bonds around 1190, 1091, and 1025 cm^−1^ related to the stretch vibration of the sulfonic acid group. The absorption peak at 1168 cm^−1^ corresponds to the C-F stretching of -CF_3_ groups, indicating that the -CF_3_ groups have been successfully incorporated into the SPIs.

**Figure 2:**
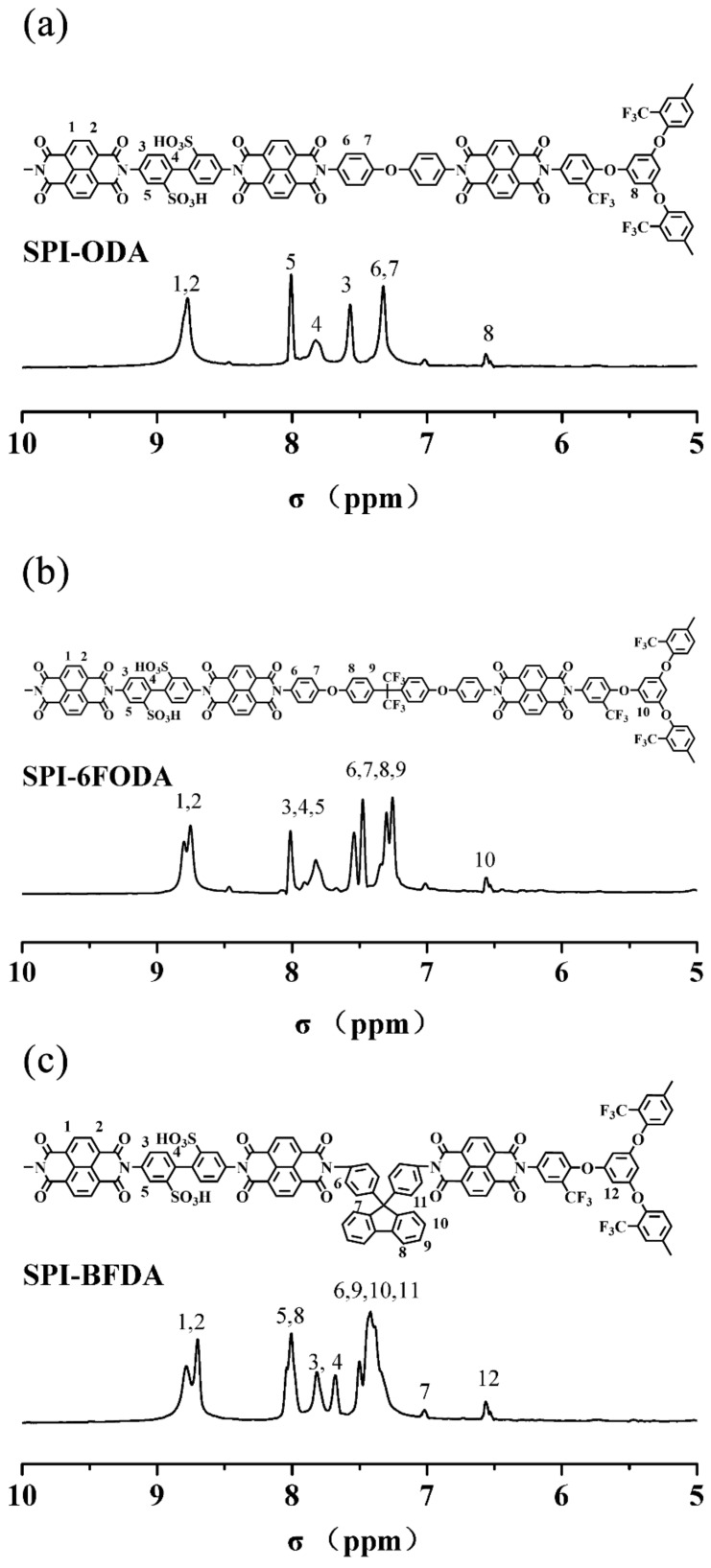
^1^H NMR spectra of the (**a**) SPI-4,4′-oxydianiline (ODA), (**b**) SPI-2,2-bis[4-(4-aminophenoxy)phenyl]hexafluoropropane (6FODA), and (**c**) SPI-4,4′-(9-fluorenylidene)dianiline (BFDA).

The ^1^H NMR spectra of three series SPIs are shown in Figure 2. The proton peaks of acid anhydride (corresponding to positions 1 and 2 in Figure 2) are always found at around 8.75–8.8 ppm. Next, the peaks of protons on the benzene ring in biphenyl sulfonic acid (corresponding to positions 3, 4, and 5 in Figure 2) are found at around 7.5–8.0 ppm. These results demonstrate the successful imidization of SPIs membranes. As shown in Figure 2a, the ^1^H NMR spectra of SPI-ODA has a single peak at 7.3 ppm (corresponding to positions 6 and 7 in Figure 2a), which is identified as the peak of protons on the benzene ring in ODA. At the same time, the proton peak around 6.5 ppm (corresponding to position 8 in Figure 2a) is assignable to the proton peak on the benzene ring in the B_3_ monomer. As shown in Figure 2b, the ^1^H NMR spectra of SPI-6FODA have a series of peaks at 7.0–7.5 ppm (corresponding to positions 6, 7, 8 and 9 in Figure 2b), which are assignable to the proton peak on the benzene ring in 6FODA. Moreover, the proton peak around 6.5 ppm (corresponding to position 10 in Figure 2b), may be the peak of the proton on the benzene ring in the B_3_ monomer. As shown in Figure 2c, the ^1^H NMR spectra of SPI-BFDA have a series of peaks at 7.0–7.5 ppm (corresponding to positions 6, 7, 8, 9, 10, and 11 in Figure 2c), which belong to the proton peak on the benzene ring in BFDA. Meanwhile, the proton peak around 6.5 ppm (corresponding to position 12 in Figure 2c) may be the proton on the benzene ring in the B_3_ monomer. All these ^1^H-NMR spectra results confirmed that three series of SPIs were successfully prepared.

**Figure 3:**
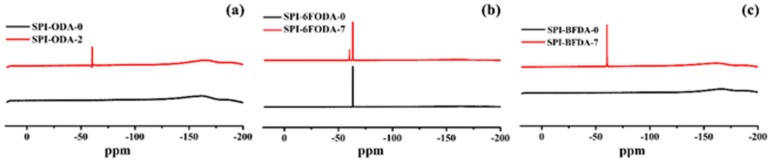
^19^F NMR spectra of the (**a**) SPI-ODA, (**b**) SPI-6FODA, and (**c**) SPI-BFDA.

The branched structures in SPIs were determined by ^19^F NMR (Figure 3). The proton peak of the B_3_ monomer in all the SPIs is found around 60 ppm, which confirmed the successful synthesis of SPIs with branched structures.

**Figure 4:**
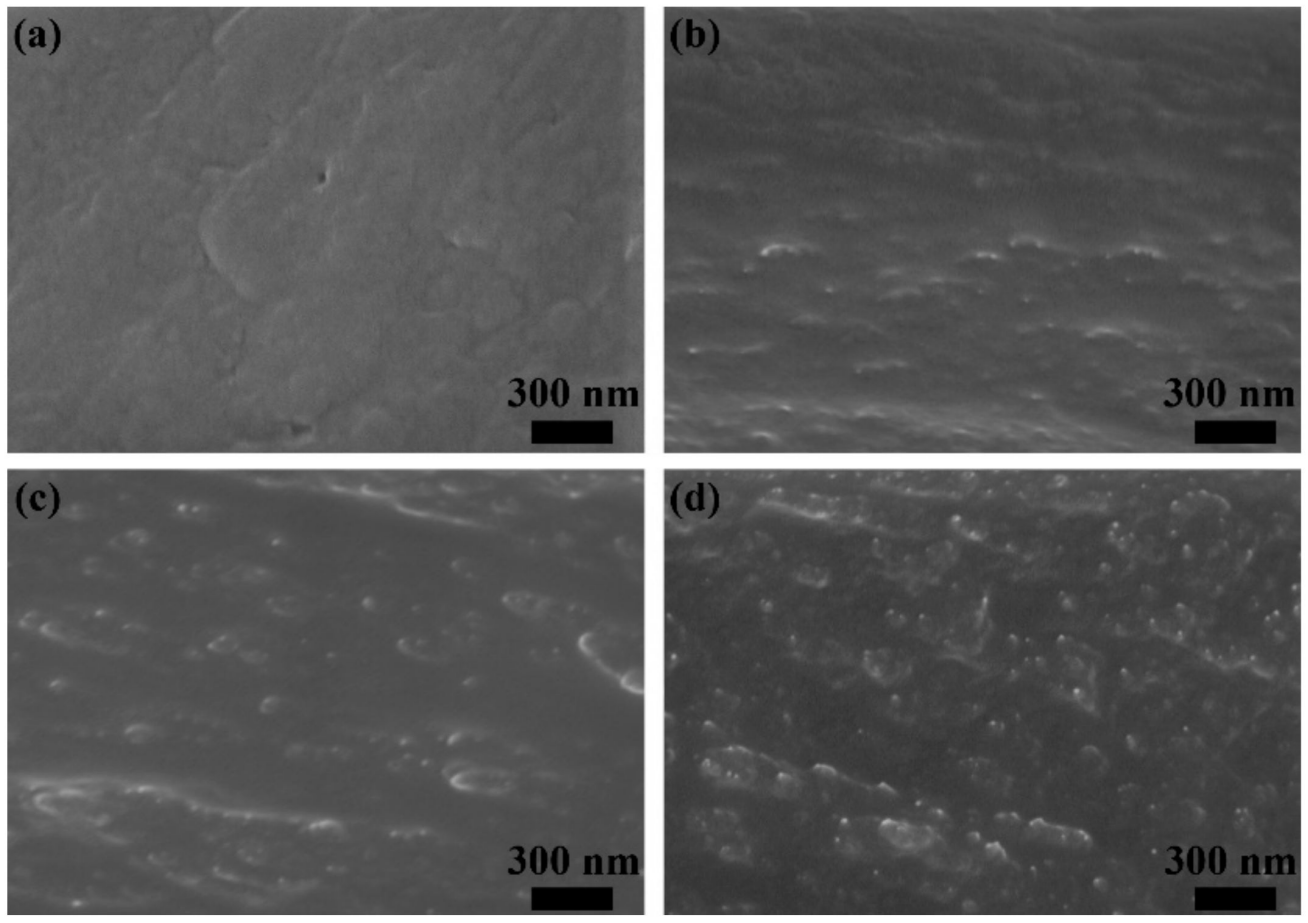
The cross section FESEM images of (**a**) SPI-BFDA-1, (**b**) SPI-BFDA-3, (**c**) SPI-BFDA-5, and (**d**) SPI-BFDA-7.

In addition, the microstructure of the representative branched SPI-BFDA-x membranes was evaluated. As shown in Figure 4, as the branching degree increased, the bumps and microcavities increased on the cross-section of the branched membranes because the unique three-dimensional structures increased, which coincided with the results reported in the literature [53].

### 3.2. Thermal and Mechanical Properties

**Figure 5:**
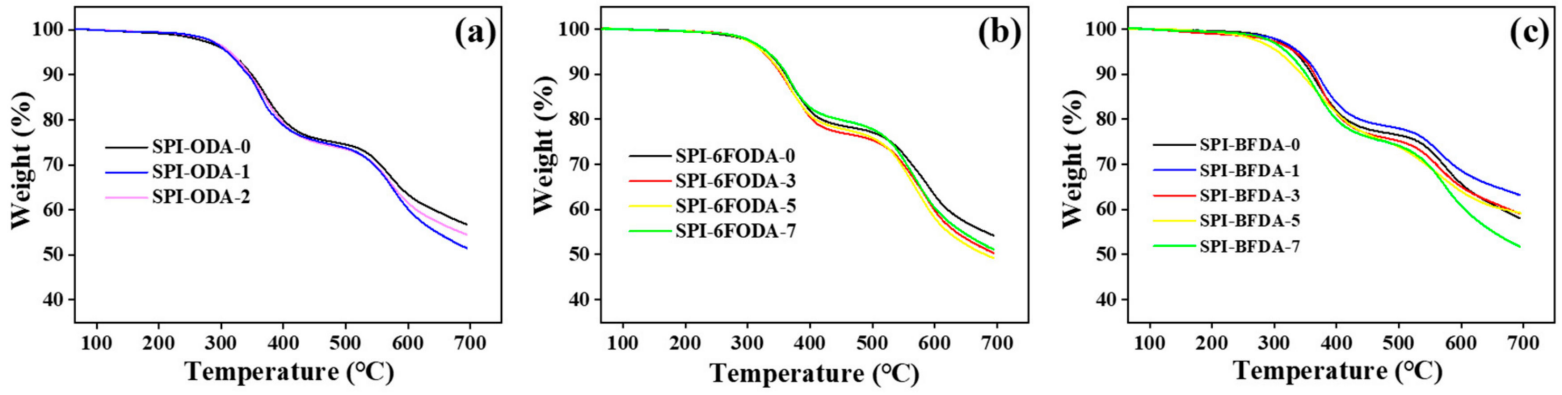
TGA curve of (**a**) SPI-ODA based membranes, (**b**) SPI-6FODA based membranes, and (**c**) SPI-BFDA based membranes.

Thermal properties of the SPIs membranes were investigated via TGA analysis under nitrogen protection. All samples were preheated at 100 °C for 20 min in the TGA furnace to remove the moisture, and the TGA curves of the three series of SPIs membranes are presented in Figure 5. All the SPIs membranes exhibited the two-step degradation pattern. The first weight loss step at about 345 °C was associated with the thermal degradation of the sulfonic acid groups [54]. The second stage weight loss started above 500 °C and was mainly due to the degradation of the polyimide main skeleton. All SPIs membranes exhibited excellent thermal stability, meeting the requirements for fuel cell preparation and application.

**Figure 6:**
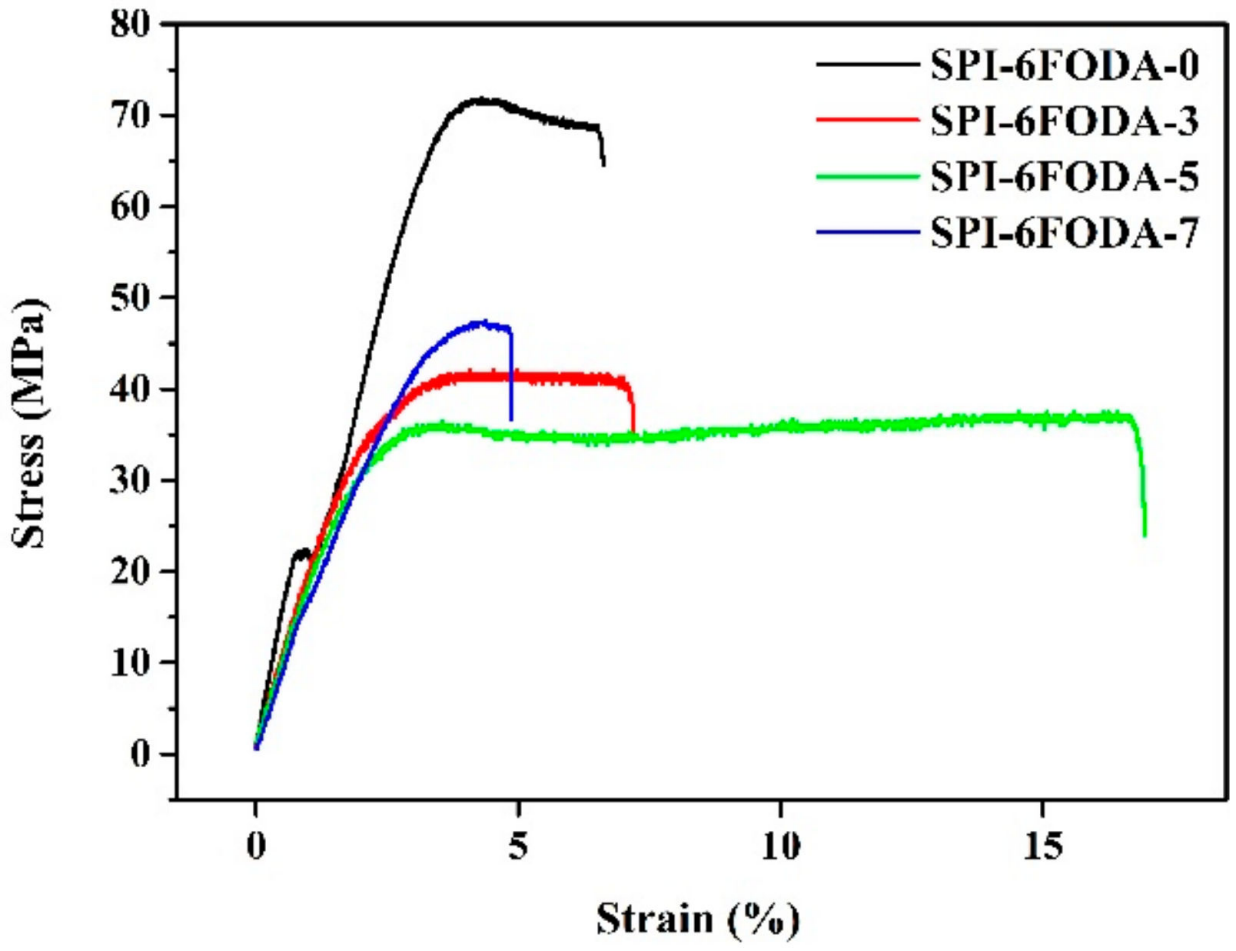
Stress–strain curves of the membranes based on SPI-6FODA.

The mechanical properties of PEM have important implications for the preparation of membrane electrodes and the durability of fuel cells. The mechanical properties of the SPI membranes are summarized in Table 2 and Figure 6. The test results show that the linear SPIs membrane had excellent mechanical strength. However, the introduction of the branched structure weakened the entanglement and interaction of the macromolecular chains. Therefore, branched SPIs have lower mechanical strength. Young’s Modulus of the typical 6FODA based membranes has been tested, which are higher than those of other proton membranes, indicating the membranes are more brittle than those in the literature [48]. The thickness and viscosity (1 mg/mL in DMSO) of the membranes are shown in Table 2. The viscosity of the branched polymers is lower than that of the linear polymer under the same testing condition, which was in accordance with reference [48].

### 3.3. Water Uptake (WU) Values and Ion-Exchange Capacity (IEC)

**Figure 7:**
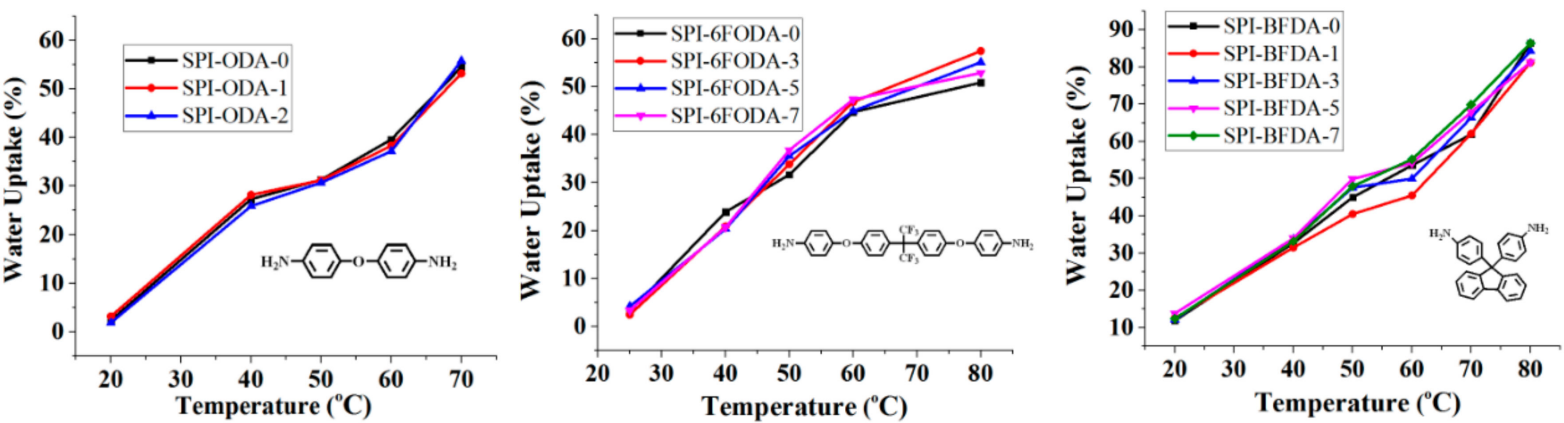
Water uptake of the three SPI based membranes.

WU is the core property of PEM, which directly affects the proton conductivity, chemical stability, dimensional stability, and mechanical properties. PEMs with high-water-uptake usually have high proton conductivity, but the mechanical properties, chemical stability, and dimensional stability of the membrane will be decreased. The WU of the PEM mainly depends on the content of the sulfonic acid group, at the same time, the molecular structure of the polymer also has a great influence on the WU of the membrane. The WU of the three series of SPIs membranes is presented in Figure 7. The results indicated that the water uptake at room temperature is very low, which can be increased by increasing the temperature. Under the same degree of sulfonation, the WU of the SPIs membranes with different non-sulfonated diamine monomers varies greatly. The SPI-BFDA series membranes introduce a large sterically hindered and kinked fluorenyl on the side chain of the molecule, which facilitates the absorption of water molecules, and thus has the highest water uptake.

Introducing the branched structure in sulfonated polyimide can increase the water absorption of the membrane. However, the increase of SPIs WU is not as obvious as sulfonated block poly(arylene ether)s [46]. This may be due to the strong interaction between the acid anhydride group and the amine group in the polyimide molecular chain, which is not conducive to the absorption of water molecules. In SPI-ODA and SPI-6FDA series membranes, due to the presence of flexible ether bonds the molecular chain is tightly bound and hinder the movement of water molecules in the molecular chain, so the branching structure is not obvious for improving WU. In SPI-BFDA series membranes, due to their larger steric hindrance on the side chain, the branching structure has a little increase in the WU of the membrane.

The theoretical IEC value of the membrane is calculated by the molecular structure, and the actual IEC value is determined by the acid-base titration. The theoretical and experimental IECs of the SPIs are shown in Table 3. The SPI-ODA series of membranes were hydrolyzed during the test, so the IEC value was not measured. The theoretical IEC values of the SPI-6FODA series of membranes are comparable to the experimental IEC values. However, in the case of SPI-BFDA, a little difference between the theoretical IEC value and experimental IEC value occurred, which may be due to the poor hydrolysis stability of SPI-BFDA membranes.

### 3.4. Chemical Stabilities

When SPIs proton exchange membranes are used in fuel cells, their chemical instability mainly comes from two aspects: (1) hydrolysis of imide rings under high humidity and high temperature conditions; (2) being attacked by peroxide and hydroxyl radicals, generated by crossover or bleeding air reacting with hydrogen in the anode [40]. In order to study the hydrolytic degradation pattern of SPIs, the hydrolytic stability of SPI_S_ membranes was tested in deionized water at 80 °C. The test results of the three series of SPIs membranes are presented in Table 4. The two series of SPI films prepared from ODA and BFDA as non-sulfonated diamine monomers have poor hydrolytic stability and quickly become brittle and broken during testing. The SPIs films prepared with 6FODA as non-sulfonated diamine monomer have good hydrolytic stability, which can still remain intact after soaking in deionized water at 80 °C for 120 h, and retain a certain degree of toughness to bend. This is attributed to the hydrophobic -CF_3_ group in the 6FODA monomer, which can reduce the contact of the water molecule with the imide ring and effectively protect the polymer backbone at the same degree of sulfonation. In SPI-BFDA series membranes, due to the introduction of a large sterically hindered fluorenyl group in the main chain, high-water uptake was shown, resulting in low hydrolysis stability of the membrane.

**Figure 8:**
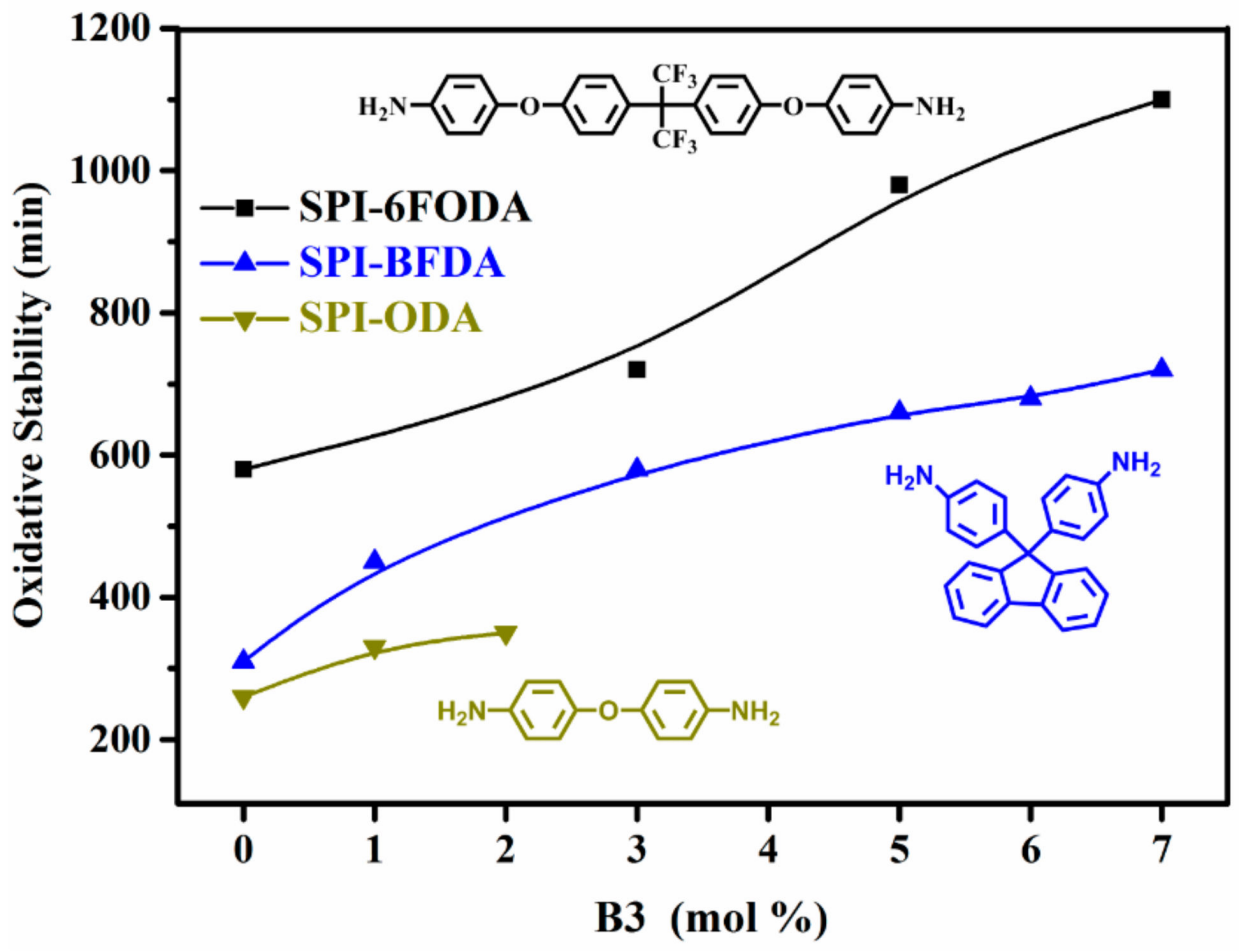
Oxidative stability of the three SPI based membranes.

In general, the oxidative stability test of PEMs was performed at 30 °C in Fenton’s reagent (30% H_2_O_2_ + 20 ppm FeSO_4_). In this condition, the main degradation pattern of the polymer will be oxidative degradation. The results of the three series of SPIs membranes are presented in Figure 8. It is obvious that the crushing time of all the films was greatly prolonged with the increase of the degree of branching. The results show that the branched structure can effectively improve the oxidative stability of SPI. This is due to the branched structure in the polymer backbone, and the branched film can remain intact even if the molecular chain is attacked by free radicals generated in the electrode.

### 3.5. Proton Conductivity

**Figure 9:**
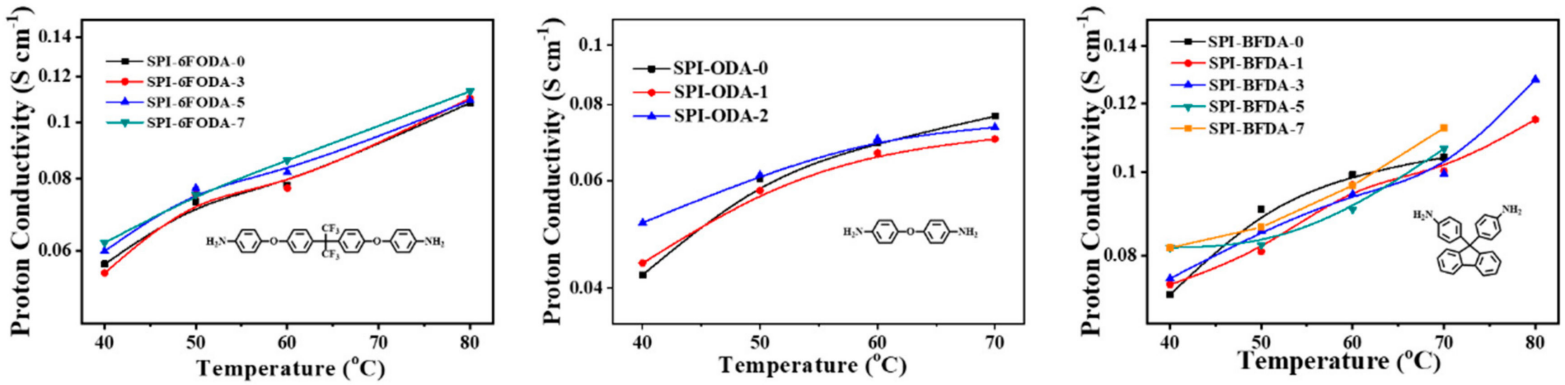
Proton conductivity of the three SPI based membranes.

PEMs with conductivity higher than 0.01 S cm^−1^ can be used in fuel cells [6,8]. The proton conductivity results of the three series of SPIs membranes are presented in Figure 9. The three series of synthesized SPIs have enough proton conductivity, meeting the requirement of the fuel cell. The proton conductivity of the membrane rises with temperature increased over the temperature range tested. The membranes of the SPI-ODA and SPI-BFDA series have poor hydrolytic stability and were easily destroyed during the test. Therefore, the conductivity of some membranes cannot be measured at 80 °C. The proton conductivity of PEM depends mainly on the water uptake and the chemical structure of the membrane. In this research, the proton conductivity of the membranes follows the same trend with their water uptake. The high-water absorption of the membranes will lead to high proton conductivity. Therefore, the membranes of the SPI-BFDA series with the highest WU may have the highest conductivity. However, the increase in proton conductivity of the branched membrane is not obvious, and the reason is the same as in the analysis of the effect on WU.

## 4. Conclusions

In summary, three series of SPIs membranes with different structures and different degrees of branching were successfully synthesized from three non-sulfonated diamine monomers (ODA, 6F0DA, BFDA). The chemical stability, WU, IEC, proton conductivity, and other physical and chemical properties were discussed. The results showed that the chemical structures have a significant effect on the chemical stability of SPIs and further affect the other physical and chemical properties of SPIs. The branched structure can greatly improve the chemical stability of SPIs membranes. Especially the introduction of branched structures in the SPI-6FDA series membranes enhanced the hydrolytic and chemical stability. At the same time, the branched structure can also improve the proton conductivity of the membrane to some extent. Therefore, the selection of chemical structures with electron donating groups and high hydrolytic stability as well as the introduction of branched structures are effective strategies for obtaining high performance SPIs used in PEMFCs.

## Figures and Tables

**Table 1:** The feed ratios and degree of branching (DB).

SPIs	Molar Ratio						DB
	NDTA	Non-Sulfonated Diamine Monomers			DAPS	B_3_	
		ODA	6FODA	BFDA			
SPI-ODA-0	3	1	-	-	2	0	0
SPI-ODA-1	3	0.97	-	-	2	0.02	1
SPI-ODA-2	3	0.94	-	-	2	0.04	2
SPI-6FODA-0	3	-	1	-	2	0	0
SPI-6FODA-3	3	-	0.91	-	2	0.06	3
SPI-6FODA-5	3	-	0.85	-	2	0.10	5
SPI-6FODA-7	3	-	0.79	-	2	0.14	7
SPI-BFDA-0	3	-	-	0	2	0	0
SPI-BFDA-1	3	-	-	0.97	2	0.02	1
SPI-BFDA-3	3	-	-	0.91	2	0.06	3
SPI-BFDA-5	3	-	-	0.85	2	0.10	5
SPI-BFDA-7	3	-	-	0.79	2	0.14	7

**Table 2:** Tensile strength, thickness, Young’s Modulus, and viscosity of three series of SPIs.

Sample	Thickness (μm)	Tensile Strength (MPa)	Young’s Modulus (MPa)	Viscosity (dL g^–1^)
SPI-ODA-0	30	62.1	-	-
SPI-ODA-1	31	62.0	-	-
SPI-ODA-2	33	50.7	-	-
SPI-6FODA-0	52	71.7	1720	0.83
SPI-6FODA-3	52	47.4	1326	0.69
SPI-6FODA-5	55	41.7	1223	0.72
SPI-6FODA-7	53	32.1	1145	0.61
SPI-BFDA-0	47	62.3	-	0.54
SPI-BFDA-1	46	66.4	-	-
SPI-BFDA-3	44	46.2	-	0.46
SPI-BFDA-5	47	41.2	-	0.49
SPI-BFDA-7	45	38.2	-	0.31

**Table 3:** Ion-Exchange Capacities (IECs), degree of branching values, and Thickness.

Polymer	Degree of Branching	IEC (mmol g^−1^)	
		Theoretical	Experimental
SPI-ODA-0	0%	-	-
SPI-ODA-1	1%	-	-
SPI-ODA-2	2%	-	-
SPI-6FODA-0	0%	2.01	1.98
SPI-6FODA-3	3%	2.01	1.91
SPI-6FODA-5	5%	1.99	1.89
SPI-6FODA-7	7%	1.97	1.90
SPI-BFDA-0	0%	2.38	1.98
SPI-BFDA-1	1%	2.36	2.08
SPI-BFDA-3	3%	2.33	1.82
SPI-BFDA-5	5%	2.31	1.82
SPI-BFDA-7	7%	2.29	2.04

**Table 4:** Hydrolytic stability of sulfonated polyimide membranes.

Membrane	Broken Time	Weight Residue after 24 h	Weight residue after 120 h
SPI-ODA-0	140 min	-	-
SPI-ODA-1	130 min	-	-
SPI-ODA-2	150 min	-	-
SPI-6FODA-0	>120 h	100%	92.8%(Flexible)
SPI-6FODA-3	>120 h	97.2%	89.2%(Flexible)
SPI-6FODA-5	>120 h	100%	90.3%(Flexible)
SPI-6FODA-7	>120 h	98.2%	87.5%(Flexible)
SPI-BFDA-0	210 min	-	-
SPI-BFDA-1	290 min	-	-
SPI-BFDA-3	270 min	-	-
SPI-BFDA-5	240 min	-	-
SPI-BFDA-7	260 min	-	-
